# Use of social media big data as a novel HIV surveillance tool in South Africa

**DOI:** 10.1371/journal.pone.0239304

**Published:** 2020-10-02

**Authors:** Alastair van Heerden, Sean Young

**Affiliations:** 1 Human and Social Development, Human Sciences Research Council, Pietermaritzburg, KwaZulu Natal, South Africa; 2 Developmental Pathways for Health Research Unit, University of the Witwatersrand, Johannesburg, Gauteng, South Africa; 3 Department of Informatics, University of California Institute for Prediction Technology (UCIPT), University of California Irvine, Irvine, CA, United States of America; 4 Department of Emergency Medicine, University of California, Irvine, CA, United States of America; University of Albany, State University of New York, UNITED STATES

## Abstract

Sub-Saharan Africa has been heavily impacted by the HIV/AIDS epidemic. Social data (e.g., social media, internet search, wearable device, etc) show great promise assisting in public health and HIV surveillance. However, research on this topic has primarily focused in higher resource settings, such as the United States. It is especially important to study the prevalence and potential use of these data sources and tools in low- and middle-income countries (LMIC), such as Sub-Saharan Africa, which have been heavily impacted by the HIV epidemic, to determine the feasibility of using these technologies as surveillance and intervention tools. Accordingly, we 1) described the prevalence and characteristics of various social technologies within South Africa, 2) using Twitter, Instagram, and YouTube as a case study, analyzed the prevalence and patterns of social media use related to HIV risk in South Africa, and 3) mapped and statistically tested differences in HIV-related social media posts within regions of South Africa. Geocoded data were collected over a three-week period in 2018 (654,373 tweets, 90,410 Instagram posts and 14,133 YouTube videos with 1,121 comments). Of all tweets, 4,524 (0.7%) were found to related to HIV and AIDS. The percentage was similar for Instagram 95 (0.7%) but significantly lower for YouTube 18 (0.1%). We found regional differences in prevalence and use of social media related to HIV. We discuss the implication of data from these technologies in surveillance and interventions within South Africa and other LMICs.

## Introduction

Ever increasing sources of digital data relevant to health, inexpensive storage, and new techniques for analysing these data offer an incredible opportunity to improve human health and wellbeing [1–3]. National Health Systems and ongoing population surveillance continue to improve as new sources of data come online. Some of the most promising technologies and approaches may include the internet of things (IoT) and cyberphysical systems (CPS), digital phenotyping through user–phone interactions, and user generated data on large social platforms such as Facebook, Instagram and Twitter [4]. These emerging “social data” sources may radically change the granularity, volume, velocity, and variety of patient health and wellness data [5]. In the next 10 years, taking these heterogenous data made up of highly dimensional sparse data and applying advanced statistics methods and novel computational approaches to machine/artificial intelligence could result in a transformation in biomedical and behavioural research.

A useful roadmap to frame this journey was proposed by Bardhan et al. [6]. It foregrounds the importance of connection, in particular of people, systems and data. They envisage a transition from provider-centred practices to patient-centred care facilitated through online health communities, artificial intelligence and sensor data. The authors note that the systems supporting population health will require the integration of multiple sources of data, such as physiological measures, genomic biomarkers, health record and potentially social media content. Finally, the connected data produced by connecting people and systems will allow diagnostic advances to be made using data dependant analytic approaches.

This plan looks exciting and feasible in high-income countries (HICs), but low- and middle-income countries (LMICs) lag behind HIC with undeveloped health systems that struggle under weight of enormous burden of disease. Further, advances made in HIC do not automatically translate through a simple process of adaption into tools that are readily useable by LMICs. For many parts of the world the biggest challenge in public health epidemiology is not connecting people, systems and data but the dearth of accurate and up to date surveillance data. Collecting, cleaning, analyzing, and making available reliable data which can be used for evidence-based planning, decision and policy making is still the priority in many parts of the world. Traditional approaches to disease surveillance lean heavily on established infrastructure and a network of data provides for reporting. The approach is costly and can result in significant delays between data capture and reporting while further burdening already strained healthcare infrastructure [7]. Without the financial resources or human capacity available in high income countries, achieving similar levels of health and disease surveillance in low- and middle- income countries (LMIC) will require novel solutions that leapfrog the public health surveillance infrastructure that is used for decision making in more well-resourced countries.

For example, rather than relying on traditional antennal clinic data to estimate HIV prevalence, another possible solution would be to explore whether the vast quantities of data generated by people (i.e., user-generated data) through their use of information and communication/social technologies might be harnessed for public health surveillance [8, 9]. These data can be broadly broken down into two primary domains, namely user content generated through active engagement and device generated data which can be passively collected. Three broad areas of user generated content can be identified. First, interacting with and posting to platforms such as Facebook, Twitter or Twitch produces information both from the content of the post (which may include text, images and/or video) as well as about social networks, user location and other paradata. Second, people produce vast amounts of data through smartphone messaging applications. These data could come either from carrier operated services such as Short Messaging Service (SMS) or through the use of messaging applications, e.g. iMessanger, WhatsApp and WeChat. Finally, user generated searches on e-commerce sites and search engines offer a third category of user generated digital data.

Passively generated data, descriptively referred to as a digital exhaust, are the data produced as a by-product of human computer interactions [10]. Examples of such data include reviewing the time a phone screen is first turned on in the morning and last turned off at night to give a sense of daily routine and hours of sleep or converting accelerometer data into a measure of daily activity [11, 12]. As devices become more deeply embedded in the environment, passive data generated by mobile phones and wearable devices will be supplemented by, for example, devices monitoring air quality, food consumed and/or time spent commuting by foot, car or public transport. Data produced by such “smart cities” may supplement data passively produced by body worn and body carried devices for health surveillance [13, 14].

A number of studies have been conducted looking at the prevalence of use of these digital data, ways that these data can be used to predict health outcomes, and ways the insights from these data can potentially be implemented by researchers and health departments [9, 15–18]. However, this work has largely been conducted in HIC, rather than LMIC, where these innovative low-cost solutions may be most needed [8, 19]. Of the two approaches (user generated content versus passive data), user generated content may offer more immediate opportunities in LMIC. Due to limited fixed line infrastructure, much of the African continent has seen rapid and widespread adoption of mobile phones [20]. The United Nation’s International Telecommunication Union (ITU) report that 4 out of every 5 people in the least-developed countries (LDCs) of the world now have access to mobile-cellular networks. While internet use remains low, around 25% of people in LDCs are expected to be online in 2020. In the more well developed African countries of South Africa (54%), Kenya (85%) and Nigeria (50%), internet use is much higher, coming close to the world average when Africa is excluded (58%) [21]. The availability of mobile phones in LMIC has seen a rapid body of evidence accumulate in the field of mHealth [22–25]. Of the mHealth literature focused on social media, to date, attention has been on its use as a tool for health promotion [26–28]. Two recent reviews of studies undertaken to assess the use of social media for disease surveillance suggest that while the current evidence base has may gaps, biases and a focus on high income countries, strengths of the approach, such as effectiveness and rapid detection of disease, suggest that further exploration is warranted [29, 30].

In this paper we aim to contribute to the literature by exploring the feasibility of using data from social technologies (with social media data as a case study) for monitoring health discussions in South Africa. Due to the burden of HIV and AIDS in South Africa we focused both on the overall number of posts made within South Africa, as well as those specific to different HIV-related topics. Little known research has explored the potential of these data in LMIC’s. This is also the first study we are aware of that explores this topic using YouTube and Instagram (which are some of the most prevalent technologies used among youth, who are at high-risk for HIV) as sources of data for public health surveillance in LMIC.

## Methods

We aimed to assesses the feasibility of our approach by assimilating a comprehensive list of potential data sources that might be used for monitoring HIV and AIDS health related discussions. We then select, as examples, three social media platforms that have large user bases in South Africa (Twitter, Instagram and YouTube) as case studies for the proposed health surveillance approach. To establish the primary social data sources available in South Africa, along with information as to the penetration and availability of data for these sources we 1) conducted a non-systematic review of the literature, and 2) undertook personal communication with the local market research companies responsible for producing the South African Social Media Landscape Report 2017 [31].

### Twitter

**Fig 1:**
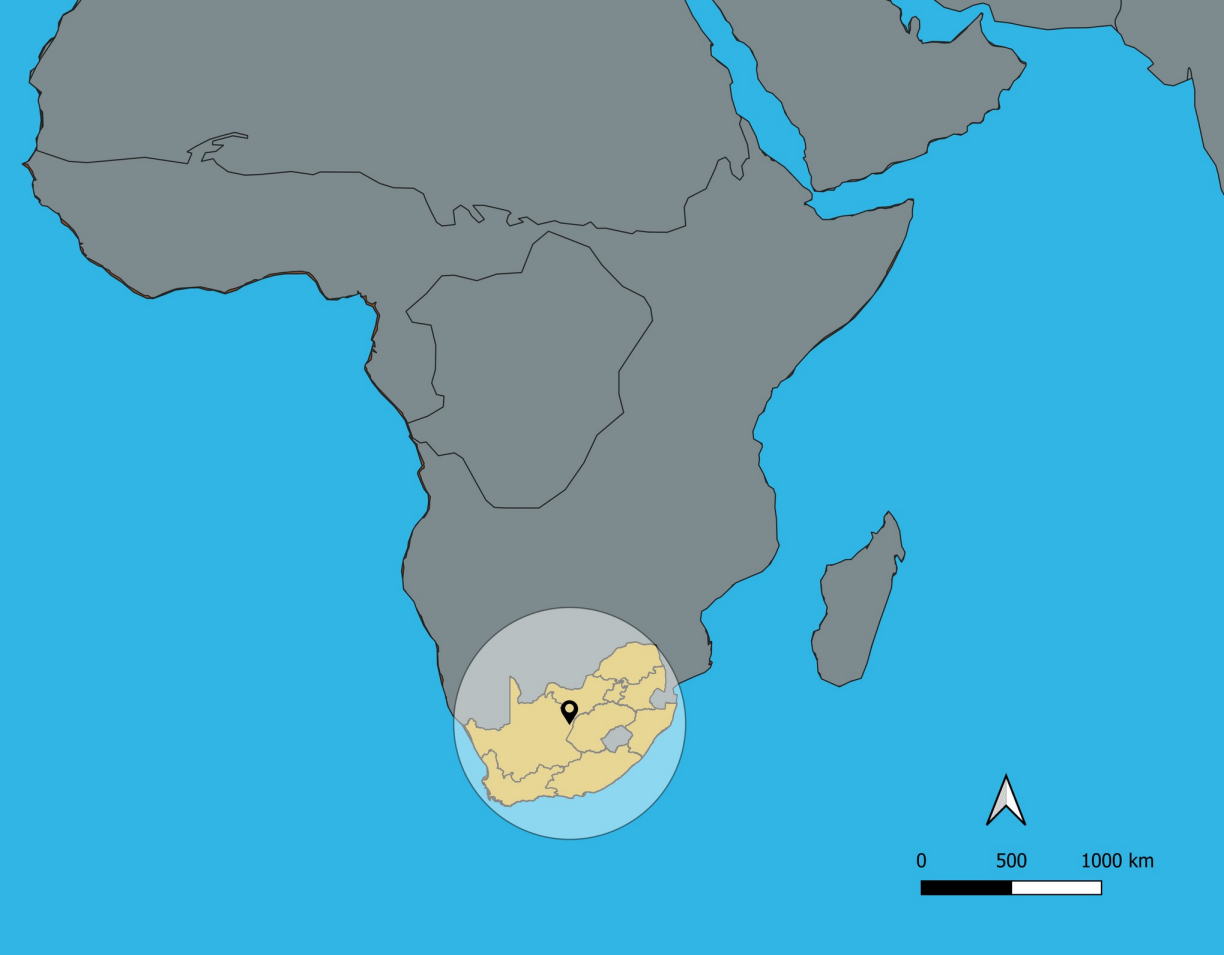
Search strategy used to identify tweets originating in South Africa.

Twitter is an Internet-based social networking and microblogging service that permits registered users to receive and broadcast very short text messages (tweets), up to 140 characters in length. Users and collective sites are identified by user names and hashtag identifiers such as “#tgif”. Messages can be resent or forwarded (retweeted), and groups of associates can associate together (as friends) in sending and receiving group tweets. As of May 2018, Twitter provides three services by which one can gain programmatic access to its global database of Tweets [32]. Firehose access is a paid for service provided by the company to select partners and is a complete export of all Tweets matching the provided filter criteria. The other two methods available are connection either to the streaming application programming interface (API) or the Search API. The streaming API returns in real-time a maximum of 1% of the total tweet volume currently available through the firehose [33]. In contrast, the Search API allows queries against an index of recent Tweets and can include filters that limit search to particular users, words and locations. The Search API is rate limited to allow only 180 queries every 15 minutes [32]. For this study the search API was used to retrieve and store Tweets. Tweets were collected in compliance with the terms of service in existence at the time. In order to perform a geographically constrained search the API, the request is accompanied by latitude, longitude and radius.[34] Using a latitude of -28.149503 and longitude of 24.543457 with a radius of 931 kilometers (578.5 miles) it was possible to construct a bounding circle that covered the entire country. Fig 1 depicts this strategy used to proximally limit Tweets to those originating from South Africa. The search API uses a two-stage process when requested to conduct a geo search.

### Instagram

Instagram is a popular photo and video-sharing social networking service. Methods for interfacing with the Instagram API are much scarcer in the literature. Where literature does exit e.g. Hochman and Schwartz [35], the focus of geospatial analysis is on only one or two cities. A primary reason for this is the Instagram API only allows for geocoded searches within a 5 kilometers radius of a given latitude and longitude. This is typically sufficient for a city but inadequate when wishing to investigate usage at a country level. Further complicating this approach was a tightening of the geosearch API during the course of this study due to the Cambridge Analytica scandal [36]. To overcome these limitations, we implemented a simple yet novel method for extracting the images for an entire country using Instagram location ids. These ids correlate to points of interest and media can be searched for in a 5-kilometer radius around the location id point. These ids are not publicly available but can be obtained through the API by searching for a place or place name. Using a list of South African city names, we obtained a list of 16,751 location ids. Due to rate limitations on the number of queries that can be processed, a random sample of roughly 10% of these location ids (1,411) were used to construct an Instagram API query using the Media/search endpoint to search for all posts within a 5 kilometer radius of this location. These data were collected in compliance with the terms of service in existence at the time.

### YouTube

**Fig 2:**
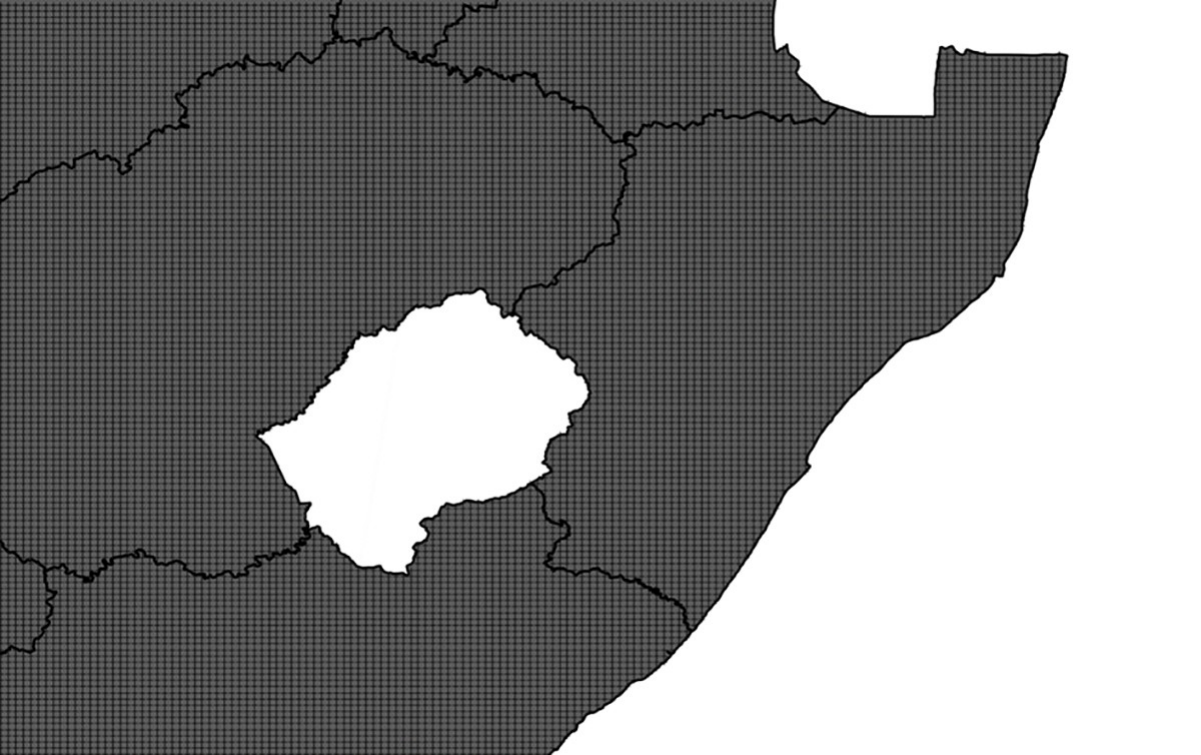
Subsection of the five kilometer point grid covering the entire extent of South Africa.

Founded in 2007 as a video sharing site, YouTube has become the second most frequently visited site on the internet [37]. An API is publicly available that can be search by geolocation. Searches are restricted to return a maximum of 500 videos with a 5-kilometer radius of the provided latitude and longitude. The 500-video limit is a function of the vast library of content that is available and a statistical search strategy used by YouTube to return the most relevant results in the shortest amount of time possible. Using Quantum GIS 2.10 software[38], a vector shape file containing the boundary of South Africa[39] was loaded. A python script, available as part of the mmqgis plugin [40], was then used to generate a point grid that covered the entire extent of South Africa. The spacing of the grid points can be specified using either the project coordinate reference system (CRS) or absolute values (meters, kilometers, feet, miles). Generated points are aligned at even multiples of the provided values. Using this grid packing approach, a list of 46,220 candidate lat/long points were generated each spaced five kilometers from each other (Fig 2).

For each lat/long point generated by the packing algorithm, it was then possible to construct a YouTube API query to search for all videos within a 5-kilometer radius of this point. The id of each video retrieved was then used to search for and return all comments attached to the video. These data were collected in compliance with the terms of service in existence at the time.

### Data processing

Access to each of the three social media sites was coded into python scripts and data stored in relational MySQL database. Keyword queries for topics related to HIV included the terms “HIV”, “AIDS”, “sex”, and “fuck”. A coding scheme was generated by the last author and all media related to these terms were coded by the middle author with the first author reviewing all coded media. Pseudo code for each of the Twitter, Instagram and Youtube python scripts are provided in S1 File.

### Analysis

Data were imported as comma separated files into SPSS v24. From these data we generated estimates of proportions, means, and confidence intervals. We then examined the data further through the use of descriptive statistics and test for differences across different geographic boundaries within South Africa and between Social Media platforms (Twitter, Instagram, YouTube) using correlations, chi-square, and analogous non-parametric tests for categorical variables, as appropriate. Consistent with the exploratory nature of this work and the relatively modest sample size, the majority of analyses relied on univariate and bivariate methods described above. Statistical significance was de-emphasized in favor of generating effect size estimates of promising associations that will be further explored in subsequent studies. Geocoded social media data were imported into QGIS 3.2 and spatial analyses undertaken. The non-parametric single variable chi-square was used to compare social media use with reference to provincial population distribution with the null hypothesis being that social media use would be equally distributed across the provinces.

### Ethics

The literature, tweets, YouTube videos and comments and Instagram posts used in this study are all publicly available and accessible to anyone with a freely available API access key. Consent for the tweet and post data to be read is secured by both companies when users sign the Twitter, YouTube and Instagram Terms and Agreements. These documents make it clear that users are agreeing to public privacy settings. This project was exempted from IRB approval as it does not meet the criteria for human subjects’ research. For the purposes of this study, user ID, names and emails were not collected or stored.

## Results

The results of the search for data sources that could be used to monitor HIV dialog for health surveillance in South Africa are presented in Table 1. Facebook had the largest number of users with 16 of the 18 million active social media users on this platform [41]. Access to geocoded data varied from simple access on platforms such as YouTube to no access (e.g. WhatsApp). A further distinction was the level of search functionality provided. Geocoded search to all media was available on platforms such as Twitter while other platforms, such as LinkedIn, limited search to known user profiles.

**Table 1:** Overall results for all platforms.

Platform	S.A. Users (millions)	Public API with geo-referenced data	Platform Description	Data Description
**Facebook**	16*	Only individual data is legally and ethically accessible	Online social media and social networking service	The Facebook Graph API is the primary mechanism for accessing data on the platform. Once authorized by the user, the API can be used to programmatically query data, post new stories, manage ads, upload photos, and perform a wide variety of other tasks.
**YouTube**	8.74*	Access to geocode search for media is available.	Video-sharing platform with user generated comment owned by Google	With the YouTube Data API, access is available to search for videos matching specific keywords, topics, locations, publication dates and comment posts.
**Twitter**	8*	Access to geocode search for media is available but mediated by access tier and cost	News and social networking service on which users post and interact with messages known as "tweets".	Offers three tiers of access which increase functionality, search volume and period of search the Twitter API provides all the functionality needed to search, filter and retrieve geocoded Tweet data.
**Linked-In**	6.1*	Location data is available for some user and company profiles	A business and employment-oriented online platform	The LinkedIn API grants access to uniform representations of people, companies, jobs, and the interactions and relationships between them.
**Instagram**	3.8*	Access to geocode search for media is available but this may change in the coming months.	A photo and video-sharing social networking service owned by Facebook	On July 31, 2018, Instagram retired the original public API in favor of the Instagram Graph API. Further restrictions to public content access are set for the 11th December 2018.
**WhatsApp**	49% of South Africans aged 18 to 64 (roughly 27 million people)**	Unknown. WhatsApp do not make it clear whether geolocation data is saved with user profiles	A multi-platform messaging and Voice over IP service owned by Facebook.	On 1 Aug 2018, WhatsApp began providing limited public access to the platform through the Business API. The primary purpose is to allow businesses a means for interacting with customers. The API will enable the ability to both send, receive and read messages with an end user.
**WeChat**	5.0^#^	OpenID is a unique encrypted WeChat ID for each user which includes city.	A multi-purpose messaging, social media and mobile payment app owned by Tencent.	Access is available, once granted by the user, to sending and receiving of messages, basic user information, and payment through the mobile wallet feature (currently mainland China only).
**Text Message**	36.1^##^	Location can be approximated through knowledge of the cell tower receiving the message.	Short Message Service (SMS) is a text messaging service offered by most mobile telecommunication companies. In South Africa MTN, Vodacom, Cell C and Telkom are the main providers.	SMS is a standardized communication protocol that enable mobile devices to exchange short text messages with one another. Access to these data are owned by the mobile operators providing the service.
**Google Maps**	N/A	Location data is available as part of the mapping API	An online mapping service that offers satellite imagery, street maps, 360° panoramic views of streets, real-time traffic conditions, and route planning for traveling by foot, car, bicycle, or public transportation.	Google maps provides programmatic access to data through a number of public APIs. Static Maps, Distance Matrix, Directions, Roads, Time Zones, Geolocation and Places information are all available via API.
**Taxify**	N/A	Location data is available in order to facilitate the dispatching of vehicles to the users location	A peer-to-peer ridesharing and transportation network company similar to Uber	An API is available that, once authorized by the user, gives programmatic access to ride requests, limited user profile and ride activity history.
**Google Search Trends**	N/A	Location data is available	A product from Google that give access to normalized search term volumes from around the world	Unofficial API for Google Trends exist that give access to keyword popularity data by location, and date.
**Pinterest**	– 1.6^^^	Location data is not available through the official API	Software designed to aid discovery and saving of online information, mainly using images.	API access is granted to Users, Boards, Pins and Sections. No formal support is provided to search for pins or boards by Geolocation.
**FB Messenger**	4^th^ most downloaded app in SA	Through “quick replies” it is possible to as the user to send their location. Availability of Geolocated data is therefore limited	A standalone messaging app and platform owned by Facebook	The available API is intended to allow developers the opportunity to create rich conversational agents (chat bots) that can send and receive messages, accept payments and help users discover and engage other users of Facebook.
**Discord**	N/A	Not currently available although the rise of location based games such as Pokemon Go have resulted in a feature request being submitted for access to users current location.	A voice-over-Internet Protocol (VOIP) application intended for use in video gaming communities. Enables text, image, video and audio communication between users in a chat channel.	The Discord API enables the creation of conversational agents (bots) that can interact with people on any given chat channel.

* The South African Social Media Landscape Report 2017 [31].

** Digital in 2018: Essential Insights into Internet, Social Media, Mobile, and Ecommerce Use around the World [41].

^#^ https://expandedramblings.com/index.php/wechat-statistics/5/

^##^ Estimated on 95% of all 18 to 65 year olds in South Africa using SMS

^^^ http://www.writescene.co.za/brand-using-pinterest/

Using the methods described above for the three case study platforms a three-week period of data collection was undertaken for each starting on May 21^st^ and ending on the 8^th^ June 2018. After cleaning the raw data returned from the three platforms API and removing retweets (repeat posts that contain no new content) and duplicate posts 654,373 tweets, 90,410 Instagram posts and 14,133 YouTube videos with 1,121 attached comments remained. Of this total 29,920 (4.6%) tweets were found to originate from points outside the boundary of South Africa. Table 2 presents these data along with a disaggregation by South African province. For comparison the most recent population estimates from the South African National Statistics council are also included. It should be noted that all data represent only a random sample of media produced on each platform during the collection period.

**Table 2:** Totals of data collected from Twitter, Instagram and YouTube between May 21, 2018 and June 8, 2018 by province.

Province	Population n (%)	Twitter n (%)	Instagram n (%)	YouTube n (%)	YT Comments n (%)
**Eastern Cape**	6,916,200 (12.6)	56,396 (8.6)	12,139 (13.4)	955 (6.8)	872 (77.8)
**Free State**	2,817,900 (5.1)	33,227 (5.1)	3,933 (4.4)	542 (3.8)	0 (0.0)
**Gauteng**	13,200,300 (24.0)	260,026 (39.7)	11,653 (12.9)	4823 (34.1)	83 (7.4)
**KwaZulu-Natal**	10,919,100 (19.9)	99,664 (15.2)	7,515 (8.3)	2027 (14.3)	13 (1.2)
**Limpopo**	5,726,800 (10.4)	39,322 (6)	9,100 (10.1)	813 (5.8)	5 (0.4)
**Mpumalanga**	4,283,900 (7.8)	32,603 (5)	5,664 (6.3)	723 (5.1)	6 (0.5)
**North West**	3,707,000 (6.7)	22,739 (3.5)	7,889 (8.7)	578 (4.1)	29 (2.6)
**Northern Cape**	1,185,600 (2.2)	4,285 (0.7)	2,597 (2.9)	770 (5.4)	55 (4.9)
**Western Cape**	6,200,100(11.3)	76,190 (11.6)	29,920 (33.1)	2902 (20.5)	58 (5.2)
**Out of Bounds**	0 (0.0)	29,920 (4.6)	0 (0.0)	0 (0.0)	0 (0.0)
**Sample Total**		654,373	90,410	14,133	1,121
**South Africa**	54,956,900	624,451	90,410	14,133	1,121

Population estimates for each province provided as a reference.

**Fig 3:**
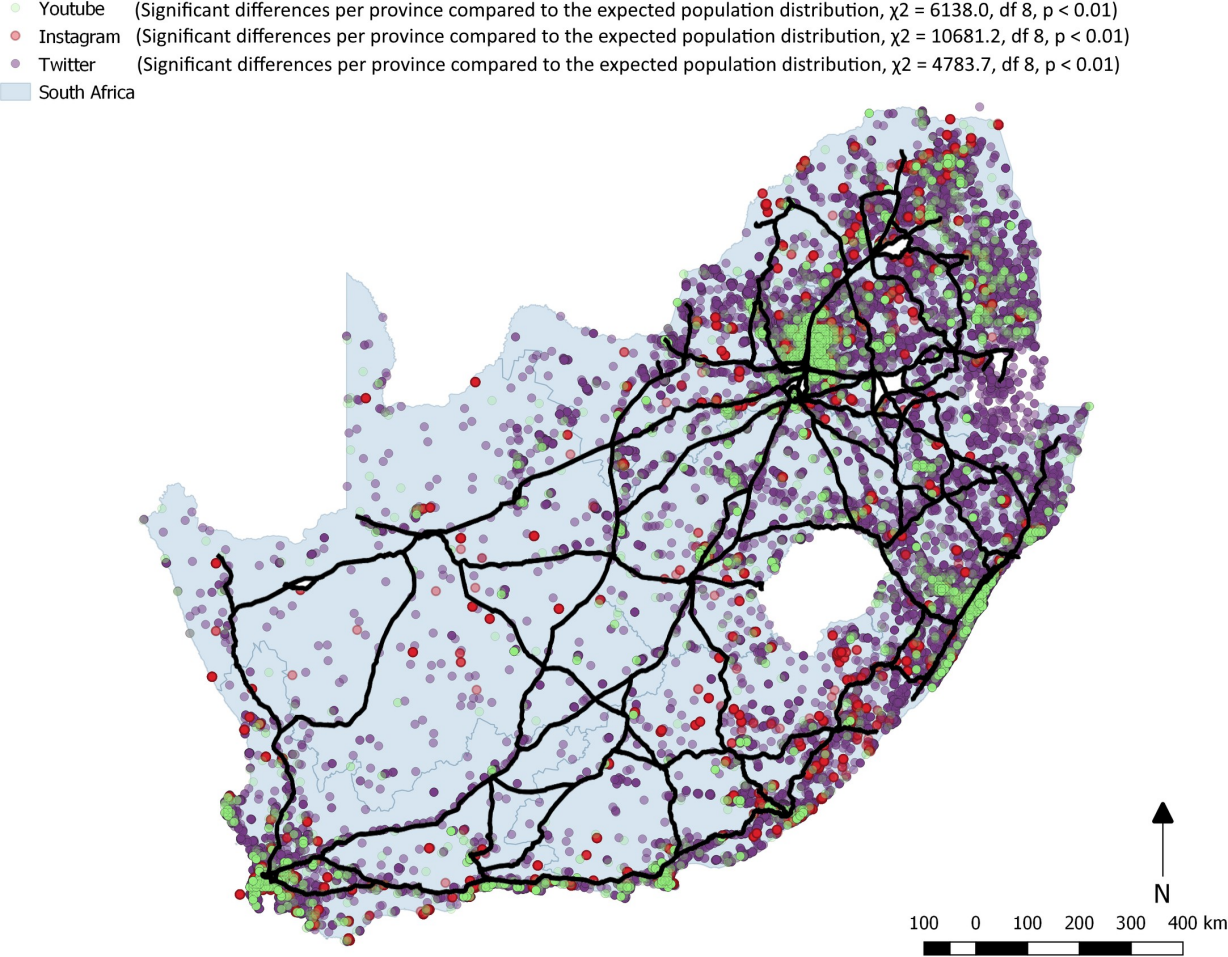
Map of South Africa showing distribution of Twitter, Instagram and YouTube social media post over a three-week period with road network overlaid.

The number of posts for each of the three platforms were unequally distributed as compared to the provincial population distribution (See Fig 3)

Social media post counts related to HIV and AIDS, retrieved using a simple keyword search, are presented in Table 3. Posts were classified using a simple coding scheme that targeted differences in media related to Risk, Risk Reduction and News / Research. These results were further disaggregated by media put out by civil society vs unaffiliated individuals. Of the 624,451 tweets, 0.7% were found to related to HIV and AIDS. The percentage was similar for Instagram (0.7) but significantly lower for YouTube (0.1%).

**Table 3:** Sexual risk and HIV related terms extracted from Twitter, Instagram and YouTube.

Topic	Twitter n (%)		Instagram n (%)		YouTube n (%)	
	NGO	Private	NGO	Private	NGO	Private
**Risk**	40 (1.0)	346 (53.9)	0 (0)	76 (80.9)	0 (0)	13 (72.2)
**Risk Reduction**	86 (2.2)	45 (7.0)	0 (0)	2 (2.1)	0 (0)	1 (5.6)
**Research / News**	2756 (71.0)	34 (5.3)	1 (100)	4 (4.2)	0 (0)	1 (5.6)
**Other / NA**	1000 (25.8)	217 (33.8)	0 (0)	12 (12.8)	0 (0)	3 (16.6)
**Total**	3882	642	1	94	0	18

Twitter–NGO: All categories are not equally represented (χ2 (3) = 128.9, p < 0.01)

Twitter–Private: All categories are not equally represented (χ2 (3) = 65.8, p < 0.01)

Instagram–Private: All categories are not equally represented (χ2 (3) = 170.0, p < 0.01)

Due to the majority of observed counts being less than 5, no analysis was performed for Instagram -NGO, YouTube-NGO or YouTube-Private

Looking at the distribution of tweets across categories reveals that NGO’s use of Twitter is significantly associated with topic (χ2 (3) = 128.9, Cramer’s V = 0.66). It appears that information sharing about the latest news and research findings is the most common use of Twitter among NGOs. For private accounts the picture is somewhat different with discussions about risk dominating the discourse. Results suggest a substantive relationship between topic and private use (χ2 (3) = 65.8, Cramer’s V = 0.47). While counts were too low to test for association in the YouTube and Instagram NGO data, a significant association was identified in the type of topic discussed by private users on Instagram (). As with Twitter, the majority of posts were related to topics of HIV risk (χ2 (3) = 170.0, Cramer’s V = 0.75).

**Fig 4:**
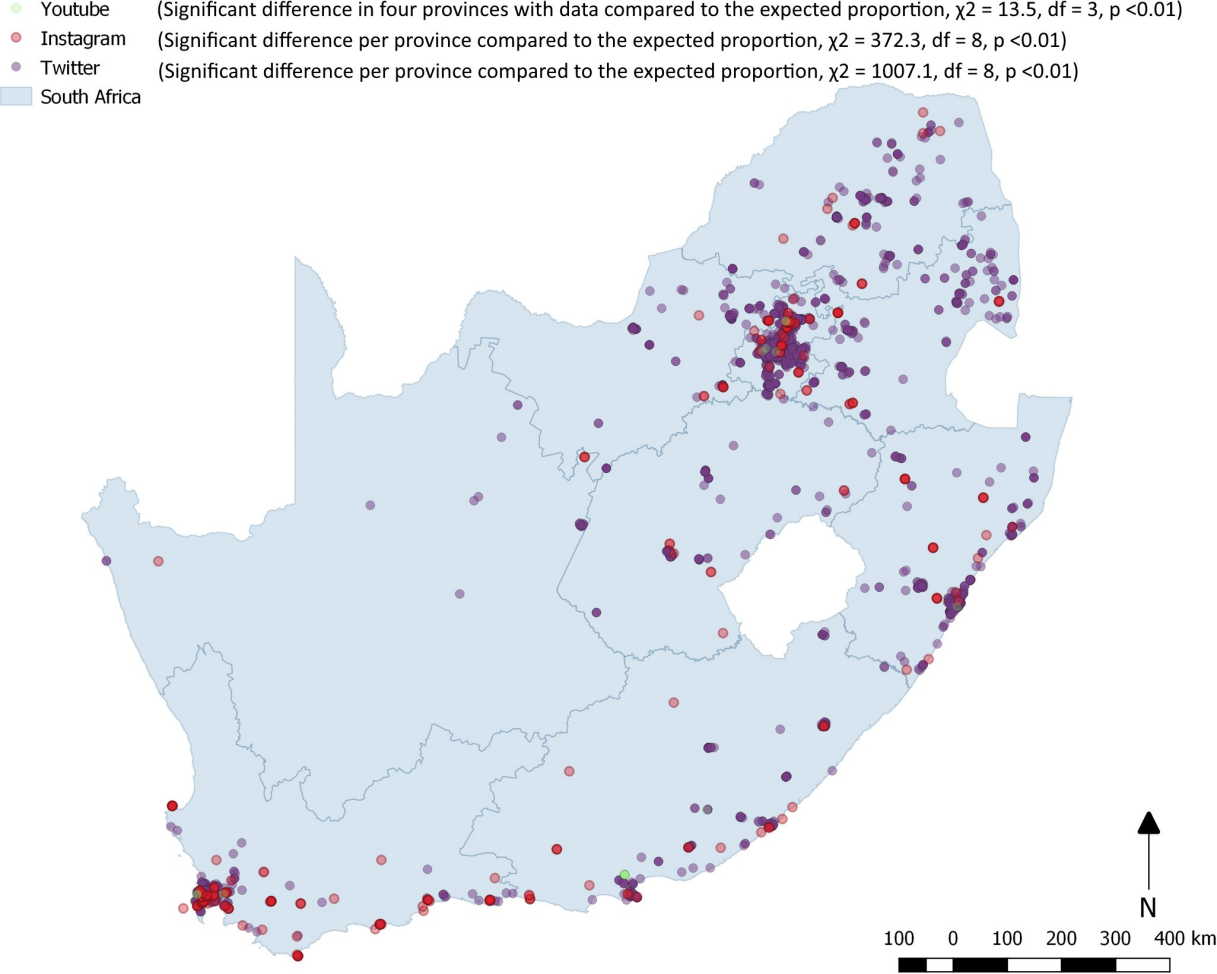
Map of South Africa showing distribution of Twitter, Instagram and YouTube social media post related to HIV over a three-week period.

Figs 3 and 4 illustrate the geographic spread of all social media post across South Africa in the three-week period (Fig 3) and for HIV related terms only (Fig 4). Compared to the provincial population proportions we found expected counts from all three social media platforms to vary significantly in their distribution. YouTube (χ2 (8) = 6138.0, p < 0.01), Instagram (χ2 (8) = 10681.2, p < 0.01), and Twitter (χ2 (8) = 4783.7, p < 0.01) were all proportionally over represented in the Western Cape. The province also has the lowest unemployment rate (20.9%) in the country (29.1%), is well known as an important tourist destination and count technology start-ups, call centres, advertising and TV production among their most important industries [42]. Twitter and YouTube are over represented in Gauteng which is the economic capital of the country, while Instagram is over represented in the North West province which is home to some of the country’s best wild life reserves. Instagram posts also overlay with the road network suggesting people taking photos while on road trips. A similar disproportionate distribution of posts was found across the three social media platforms in relation to provincial population distribution. Gauteng and the Western Cape accounted for 35.3% of the population and 66.1% of all Instagram, 59.4% Twitter and 8 of the 18 YouTube videos and posts.

## Discussion

Twitter, Instagram and YouTube were all found to have a significant number of active users in South Africa. While the majority of posts emanate from users in the large metros of Cape Town, Johannesburg and Durban, activity is visible across the country. This is particularly true of Twitter, found to be the most popular of the three platforms, and Instagram. Over half of all YouTube activity is isolated to the Western Cape and Gauteng. YouTube comments are unexpectedly skewed towards the Eastern Cape. Investigation suggests that the reason behind this distribution is that a video title “We can't go on like this | South Africa” received 867 comments. The video dealt with Racism in South Africa. A number of tweets were found to reference content produced outside of South Africa. The reason for these data anomalies was the search strategy used to collect tweets. A single central latitude and longitude was use and all tweets within a 931km radius stored. This meant that the dataset included tweets from neighboring countries including Botswana, Namibia and Mozambique.

Due to the burden of disease posed by HIV and AIDS, the study focused on the presence of social media posts that directly reference the disease or allude to sexual activity. Using a simple keyword search strategy under 1% of posts across all three platforms were found to be relevant. A more nuanced approach may yield better results. For the posts that were found, it becomes clear that civil society is currently only engaging with the South African community though Twitter. Around 61% of all tweets were research or news articles tweeted by Non-Governmental Organizations (NGO). Only a single Instagram post from the mothers2mothers NGO relating to HIV and AIDS was found on Instagram and no YouTube videos could be identified as being posted by similar groups. These findings suggest a clear opportunity exists for NGOs and civil society to move some of their advocacy and engagement work onto these two other platforms. Due to its size, Instagram should be prioritized. When considering the textual content of media posted by private users, sexually risky behaviors is by far the most common classification. Between 54% and 81% of posts across the three platforms fall into this category. Only a small percent (2.1%-7%) related to safer sex practices. These findings suggest that public health messages may not yet have altered people’s perceptions of HIV and the risk associated with less safe sex practices.

### Connecting people

We found that how NGOs use these three platforms to connect with people is very different from how people use the platforms to connect with each other. First, although across all platforms, Twitter had the highest frequency of posting, within platforms, the most frequent posts were “risk-related” posts, such as individuals describing their interests and/or intentions to engage in sexual risk behaviors. Further research can explore the potential of social media posts about risk to identify trends in risk and improve surveillance. Relatedly, within Twitter, NGO’s are primarily using Twitter to discuss news and risk-reduction (e.g., importance of using condoms), while individuals are using it to discuss risk. Understanding how different groups and individuals use social media sites can help inform HIV and health promotion efforts more broadly, as well as help HIV and public health outreach organizations better understand which social media sites are already saturated in order to turn their approaches to new technologies and methods.

### Connecting with data

Working with social media data can be challenging due to data volume and storage, velocity of data creation, the variety of social media data that exists and uncertainty about data quality [43]. Similar issues were encountered in this study with data volume and storage quickly exceeding the funds set aside for the study. A document, rather than relational database was used to successfully manage the unstructured nature of the collected data and traditional exploratory data analysis was found adequate for identifying missing, incomplete or outlier data. The challenge working with these unstructured data is magnified when looking to link data with structured electronic health records. Standards such as the Fast Healthcare Interoperability Resources (FHIR) provide a modern scaffolding for exchanging healthcare information electronically [44].

While access is more difficult due to a lack of a public API, we found that Whatsapp and Facebook were the two largest social media platforms in use in South Africa. Whatsapp has functionality which enables individuals to export chats as raw text. This is potentially an interesting additional source of data and might support further understanding of how health related risks and behaviors are discussed within a peer network. Despite the promise these and other social media data sources hold, it is an ongoing debate as to if, and how best these data can support the greater good while respecting the individuals rights to privacy and anonymity [45].

### Connecting with systems

The value of these non-traditional data sources to complement health systems data is becoming increasingly obvious, the National Institutes of Health initiative focusing on Data Science for Health Discovery and Innovation in Africa Research Hubs, being one example [46]. They note that infrastructure, specifically mobile phone networks, continue to grow rapidly in Africa offering a valuable opportunity to the continent to explore new sources of health data with contextually relevant ethical, legal and social implications key research areas. While the ethical issues with using social media data are becoming more well understood, for instance privacy, anonymity, and consent, their application to populations with lower level technological health literacy requires attention [47, 48].

### Limitations

The primary limitation of this study was the short period of time the social network data were collected. Funding constraints prevented data being collected for a longer period of time. Const associated with this type of data collection include the downloading and storage of large amounts of data. It is not clear how representative the months’ worth of collected data is of general usage trends and an extended data collection period would be an essential next step. A second limitation, related more to the approach than to this particular study, are the biases inherent in different forms of health data. For example, what people search for, official CDC statistics and what the media presents in terms of health and disease are likely to produced different results. Finally, we used a coding scheme to identify topics discussed on social media. This was a primitive initial method to explore differences in topics, however, more advanced methods of identifying topics and discussions are available and should be explored.

### Future directions

Image and video data collected from Instagram and YouTube present a novel opportunity for public health research. Future work should consider how best these audio-visual data could be analyzed to provide information on the health of the population. Many of the posted Instagram images include either people and/or food. Together they may offer an interesting approach to developing National models of food, alcohol and sugary drink consumption and estimates of Overweight and Obesity. Comparing National HIV surveillance data to the spread of social media posts could also yield information about the usefulness of these data for HIV surveillance and intervention. Another promising direction this work could take would be to explore the possibility of associating social media data with longitudinal data collected at country level demographic surveillance sites (DSS). With only a small additional burden on existing DSS data collection systems, it might be possible to supplement longitudinal health records of individuals living in the area under surveillance with social media data which might provide a different perspective and insight into the health risks, behaviors and conversations people are having about health thought their online profiles.

## Conclusion

Smartphones, the social media platforms people access on these devices and the content they create all hold potential as sources of health data. While feasible to collect, there is still much work required to create models that are unbiased, accurate and useful as tools for health surveillance in low and middle-income countries.

## Supporting information

S1 File (DOCX)
