# Supplementary material for: Use of social media big data as a novel HIV surveillance tool in South Africa
Source: PLoS One. 2020 Oct 2;15(10):e0239304. doi: 10.1371/journal.pone.0239304 (PMC7531824; doi:10.1371/journal.pone.0239304)
Supplement: S1 File — (DOCX) [file pone.0239304.s001.docx]

**Tweet Script**

import tweepy

import sys

import os

api = tweepy.API(auth, wait_on_rate_limit=True,

wait_on_rate_limit_notify=True)

if (not api):

print("Can't Authenticate")

sys.exit(-1)

from pymongo import MongoClient

client = MongoClient()

client.connect()

latitude = 51.474144 # geographical centre

longitude = -0.035401 # geographical centre

max_range = 5 # search range in kilometres

new_tweets = api.search(q=searchQuery, geocode="%f,%f,%dkm" % (

latitude, longitude, max_range), count=tweetsPerQry, max_id=last_id)

for tweet in new_tweets:

tweet_data = tweet._json

result = db.insert(data_db)

except tweepy.TweepError as e:

print("some error : " + str(e))

print("Downloaded {0} tweets, Saved to {1}".format(tweetCount, fName))

**Instagram Script**

import requests

import json

def get_access_token():

req_params = {"client_id": client_id, "client_secret": client_secret,

"redirect_uri": redirect_uri, "grant_type": grant_type, "code": code, "scope" : "public_content"}

request_url = "https://api.instagram.com/oauth/access_token"

r = requests.post(request_url, data=req_params)

data = json.loads(r.text)

access_token = str(data["access_token"])

return access_token

def media_search(lat, lng, access_token):

url = "https://api.instagram.com/v1/media/search?lat="+lat + \

"&lng="+lng+"&distance=5000"+"&access_token="+access_token

r = requests.get(url)

resp = json.loads(r.text)

if resp['data']:

f_name = str(location) +'.csv'

with open(f_name, 'wb') as f:

f.write(resp['data'])

f.close()

lat = '51.474144' # geographical centre of search

lng = '-0.035401' # geographical centre of search

access_token = get_access_token()

media_search(lat, lng, access_token)

**Youtube**

import pymongo

import requests

import time

import json

from pymongo import MongoClient

import xlrd

client = MongoClient()

db = client[‘youtube']

postDB = db.posts

main_url = 'https://www.googleapis.com/youtube/v3/search'

result_data = read_excel_all_columns('pointlist.xlsx')

for data in result_data:

lat = str(data[0])

lon = str(data[1])

main_params = dict(

part='snippet',

location=lat + ',' + lon,

locationRadius='5km',

type='video',

maxResults=50,

key='XXXXXX')

resp = requests.get(url=main_url, params=main_params)

youtube = resp.json()

try:

for index, item in enumerate(youtube['items']):

postDB.insert_many(item)

except:

print('No results for ' + str(lat) + ',' + str(lon))
